# CCAR2/DBC1 and Hsp60 Positively Regulate Expression of Survivin in Neuroblastoma Cells

**DOI:** 10.3390/ijms20010131

**Published:** 2019-01-01

**Authors:** Wootae Kim, Jaewook Ryu, Ja-Eun Kim

**Affiliations:** 1 Department of Biomedical Science, Graduate School, Kyung Hee University, Seoul 02447, Korea; mubear@khu.ac.kr (W.K.); busterray@naver.com (J.R.); 2 Department of Pharmacology, School of Medicine, Kyung Hee University, Seoul 02447, Korea

**Keywords:** CCAR2, Hsp60, survivin, apoptosis, survival, neuroblastoma

## Abstract

CCAR2 (cell cycle and apoptosis regulator 2) controls a variety of cellular functions; however, its main function is to regulate cell survival and cell death in response to genotoxic and metabolic stresses. Recently, we reported that CCAR2 protects cells from apoptosis following mitochondrial stress, possibly by co-operating with Hsp60. However, it is not clear how CCAR2 and Hsp60 control cell survival and death. Here, we found that depleting CCAR2 and Hsp60 downregulated expression of survivin, a member of the inhibitor of apoptosis (IAP) family. Survivin expression in neuroblastoma tissues and human cancer cell lines correlated positively with expression of CCAR2 and Hsp60. Furthermore, high expression of CCAR2, Hsp60, and survivin was associated with poor survival of neuroblastoma patients. In summary, both CCAR2 and Hsp60 are required for expression of survivin, and both promote cancer cell survival, at least in part, by maintaining survivin expression. Therefore, CCAR2, Hsp60, and survivin are candidate tumor biomarkers and prognostic markers in neuroblastomas.

## 1. Introduction

Cell cycle and apoptosis regulator 2 (CCAR2), formerly known as deleted in breast cancer 1 (DBC1), is an emerging key regulator of multiple cellular functions. CCAR2 mediates positive and negative regulation of several transcription factors, including ERα/β, GR, TR, AR, Rev-Erbα, and BRCA1, and affects transcription, metabolism, circadian cycles, and aging [1,2,3]. CCAR2 also plays a role in epigenetic modification by regulating HDAC3 and Suv39h1 [4,5]. In addition, the CCAR2-containing DBIRD (DBC1-ZIRD) complex controls alternative mRNA splicing and transcriptional elongation [6]. Other well-known functions of CCAR2 include regulation of cell death and survival. CCAR2 increases cell death in a SIRT1-dependent manner by inhibiting its deacetylase activity in response to etoposide, ionizing radiation and hydrogen peroxide [7,8,9]. By contrast, CCAR2 exerts a cytoprotective effect following ultraviolet irradiation and treatment with rotenone [10,11]. In the absence of exogenous insults, CCAR2 might also act as either a promoter or suppressor of cell survival. CCAR2 knock-out mice develop spontaneous tumors, indicating a potential role of CCAR2 as a tumor suppressor. CCAR2 knockout mouse embryonic fibroblasts (MEFs) show faster proliferation and colony formation than wild-type MEFs. By contrast, CCAR2-deficient cancer cells grow slowly, suggesting its role as a promoter for tumor cell survival [12,13]. Therefore, the role of CCAR2 in cell death and survival may depend on the context, particularly in terms of cell type and stimulus type.

Recently, we showed that CCAR2 interacts with Hsp60 in mitochondria [11]. The results of our study suggest that this interaction may contribute to the survival of neuroblastoma cells following rotenone-induced mitochondrial stress. That report was the first to show that a CCAR2-associated complex is involved in its pro-survival effects; by contrast, CCAR2-SIRT1 interaction mediates pro-apoptotic effects [7].

Hsp60 mediates pro-survival and pro-apoptotic effects via numerous Hsp60-interacting proteins, including survivin [14], pro-caspase-3 [15,16,17], hepatitis B virus X protein (HBx) [18], cyclophilin D [19], p53 [14], and Bcl-2 family members such as Bax, Bak, Bcl-xL, and Bcl-2 [20,21,22,23]. However, Hsp60 favors cell survival rather than cell death [24]. Indeed, Hsp60 homogeneous knockout in mice leads to embryonic lethality, and Hsp60 knockdown in tumor cells induces apoptosis and inhibits growth [14,25,26]. However, the common protein with which CCAR2 and Hsp60 interact to regulate cell survival has not been identified.

Survivin, one of Hsp60-interacting proteins, is a member of the inhibitor of apoptosis (IAP) family [27]. Hsp60 helps to stabilize mitochondrial survivin [14]. Survivin mediates anti-apoptotic effects by binding to several apoptosis-regulating factors, ultimately inhibiting caspases [28]. Homogeneous knockout of the survivin gene causes embryonic lethality in the animal model, and downregulation or inactivation of survivin retards tumor growth in the cell model [27,29,30].

Neuroblastoma is a common pediatric tumor that is usually diagnosed after 18 months of age, at which time it has usually metastasized. Neuroblastoma is genetically heterogeneous [31]. In particular, the unfavorable outcome of neuroblastoma is associated with deletion of chromosomes 1p or 11q, gain of chromosome 17q, or amplification of the MYCN proto-oncogene [32,33]. Survivin is mapped to chromosome 17q25, a region that is gained frequently at the advanced stages of neuroblastoma [34]. High expression of survivin correlates with the advanced stage and MYCN amplification in neuroblastoma [35,36,37]. While the association between survivin and neuroblastoma has been studied, less is known about the role of CCAR2 and Hsp60. In addition, although we reported previously that CCAR2 and Hsp60 act cooperatively to increase the survival of neuroblastoma cells [11], the underlying mechanism is unclear. Here, we show that CCAR2 forms a complex with Hsp60 and survivin, and that both CCAR2 and Hsp60 are important regulators of survivin expression. The results of current study will shed light on the mechanisms by which CCAR2 and Hsp60 regulate cell survival.

## 2. Results

### 2.1. The CCAR2-Hsp60 Complex Binds the Anti-Apoptotic Protein Survivin

**Figure 1:**
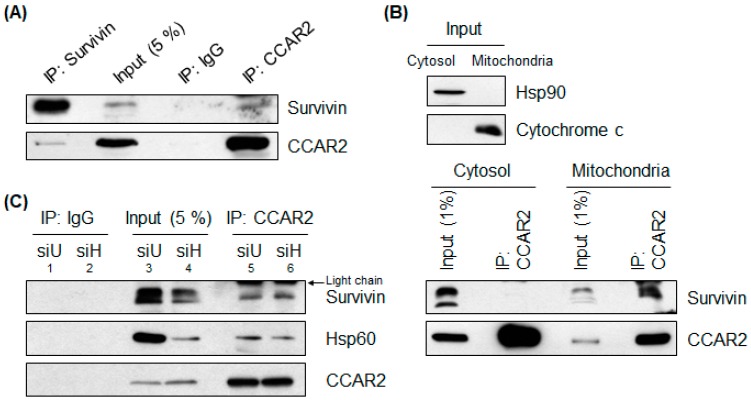
CCAR2 binds Hsp60 and survivin. Interaction between CCAR2 and survivin was examined in SH-SY5Y or HEK293 cells by co-immunoprecipitation with either an anti-CCAR2 or an anti-survivin antibody, followed by western blotting. (**A**) Interaction between CCAR2 and survivin in whole cell lysates from SH-SY5Y cells was examined. (**B**) Interaction between CCAR2, survivin, and Hsp60 in cytosolic and mitochondrial fractions isolated from HEK293 cells was examined. (**C**) SH-SY5Y cells were depleted of Hsp60 and the interaction between CCAR2 and survivin was examined. siU, universal siRNA; siH, Hsp60 siRNA.

A growing body of evidence suggests that CCAR2 and Hsp60 function as pro-survival factors; therefore, it is important to investigate how CCAR2 and Hsp60 react to cellular stress. Hsp60 interacts with several apoptosis regulators in mitochondria to inhibit apoptosis; these regulators include survivin, an anti-apoptotic protein [14]. Recently, we reported that CCAR2 is localized to mitochondria and interacts with Hsp60 [11]. Therefore, we investigated whether CCAR2 forms a complex with any of Hsp60-interacting mitochondrial proteins. We found that CCAR2 interacted with survivin in SH-SH5Y neuroblastoma cells (Figure 1A). In addition, we immunoprecipitated cytosolic and mitochondrial fractions from HEK293 embryonic kidney cells and confirmed the localization of the CCAR2-survivin complex. Although CCAR2 and survivin were detected in both the cytosol and mitochondrial fractions, the CCAR2-survivin complex was detected only in mitochondria (Figure 1B) (See Discussion section). Next, we examined the interaction between the CCAR2-Hsp60 complex and survivin. CCAR2 interacts with Hsp60 and survivin in SH-SY5Y cells (Figure 1C, lane 5). Although the expression of survivin decreased in Hsp60-depleted cells, the interaction between CCAR2 and survivin still occurred in the absence of Hsp60 (Figure 1C, lane 5 vs. lane 6), indicating that CCAR2 is a core protein involved in sequestration of survivin. Surprisingly, although Hsp60 was depleted, the extent of the interaction between CCAR2 and Hsp60 was similar in control and Hsp60-depleted cells (Figure 1C, lane 5 vs. lane 6) (See Discussion section). Therefore, these results suggest that the CCAR2-Hsp60 complex acts as a pro-survival factor via its ability to regulate binding to survivin, an anti-apoptotic protein.

### 2.2. Both CCAR2 and Hsp60 are Required for Expression of Survivin

**Figure 2:**
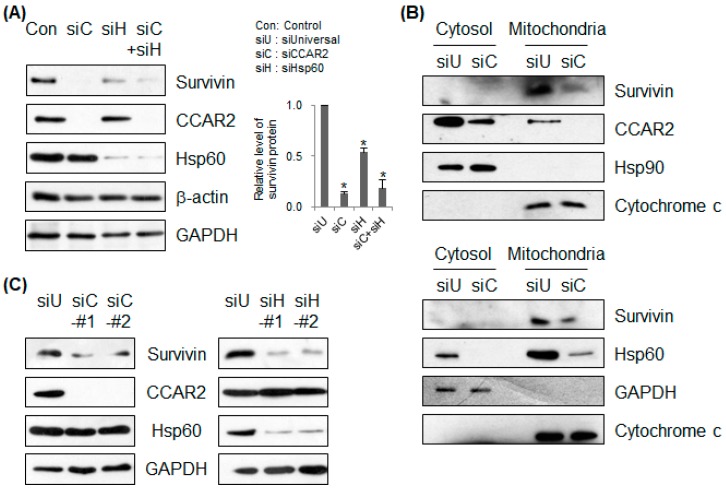
Deficiency in CCAR2 or Hsp60 reduces expression of survivin. SH-SY5Y cells were transfected with Universal (siU), CCAR2 (siC), or Hsp60 (siH) siRNA. Forty-eight hours later, expression of survivin protein was examined by western blotting. (**A**) Survivin expression was detected in whole cell lysates. The relative level of survivin protein is presented as the mean ± standard error of the mean (SEM) (*n* = 3). Asterisks (*) denote statistically significant differences (*p* < 0.05, one-way ANOVA). (**B**) Cytosolic and mitochondrial fractions were isolated to determine localization and expression of survivin. (**C**) Two different siRNAs specific for CCAR2 and Hsp60 were used to knock down their expressions.

A previous report shows that Hsp60 is required for expression of mitochondrial survivin and inhibition of apoptosis [14]. The results also show that Hsp60 deficiency results in downregulation of survivin (Figure 1C, lane 4). In addition, our previous findings demonstrate that CCAR2 deficiency renders cells more susceptible to apoptosis [11]. Therefore, we asked whether CCAR2, one of several mitochondrial Hsp60-binding partners, also affects survivin expression. SH-SY5Y cells were transfected with siRNA targeting either CCAR2 or Hsp60 (Figure 2A–C). Loss of CCAR2 or Hsp60 expression led to a significant decrease in expression of survivin protein (Figure 2A). To confirm whether CCAR2 and Hsp60 affect survivin levels in mitochondria, we isolated the mitochondrial fraction from CCAR2- and Hsp60-deficient cells. As reported previously [11], we found that CCAR2 and Hsp60 were localized to mitochondria (Figure 2B). Mitochondrial survivin was downregulated in both CCAR2- and Hsp60-deficient cells (Figure 2B). In addition, we used two different siRNAs specific for CCAR2 and Hsp60 to confirm downregulation of survivin (Figure 2C). These data indicate that both CCAR2 and Hsp60 are required for maintenance of survivin expression. However, the importance of the CCAR2-Hsp60 complex in mitochondria for maintenance of survivin needs further study (see Discussion section).

### 2.3. CCAR2 and Hsp60 Regulate Expression of Survivin mRNA

**Figure 3:**
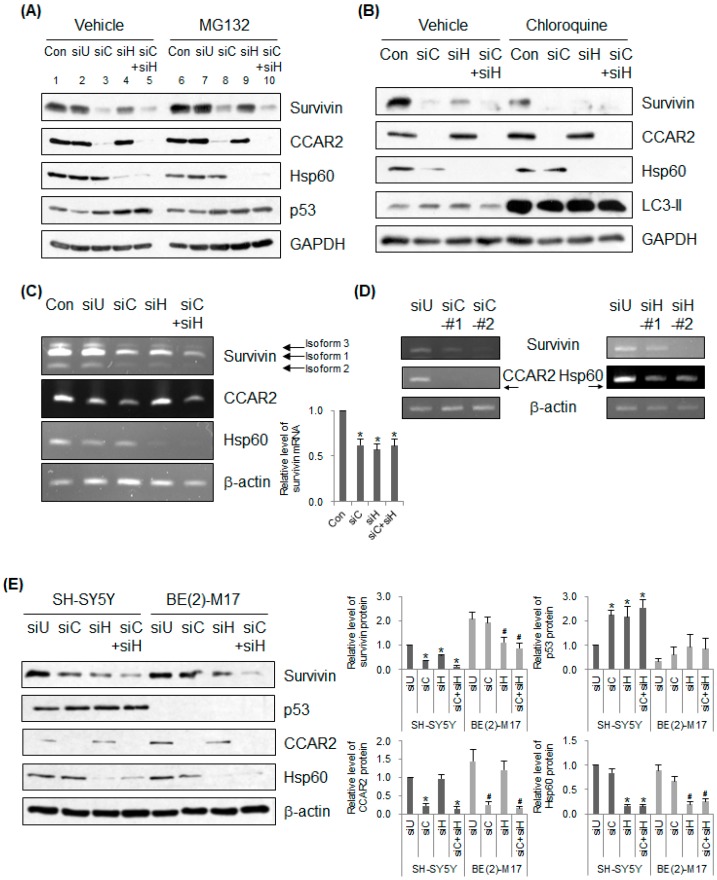
Deficiency of CCAR2 or Hsp60 reduces expression of survivin mRNA. SH-SY5Y (**A**–**E**) or BE(2)-M17 cells (**E**) were transfected with Universal (siU), CCAR2 (siC), or Hsp60 (siH) siRNA. (**A**,**B**) Forty-eight hours later, the level of survivin protein was examined by western blotting. Cells deficient in CCAR2 and Hsp60 were treated with 25 μM MG132 (**A**) or 100 μM chloroquine (**B**) 4 h or 24 h prior to cell lysis, respectively. (**C**,**D**) The level of survivin mRNA in each group of siRNA-transfected cells was measured by RT-PCR. (**C**) Levels were normalized against β-actin and quantified using ImageJ software. The relative level of survivin mRNA is expressed as the mean ± standard error of the mean (SEM) (*n* = 3). Asterisks (*) denote statistically significant differences (*p* < 0.05, one-way ANOVA). (**D**) Two different siRNAs targeting CCAR2 and Hsp60 were used to knock down their expressions. (**E**) The level of each protein was examined by western blotting. The relative level is expressed as the mean ± standard error of the mean (SEM) (*n* = 3). Indicators (*, #) denote statistically significant differences from the corresponding control cells (*p* < 0.05, one-way ANOVA).

The next question we asked was how do CCAR2 and Hsp60 control survivin expression? First, to investigate whether depleting CCAR2 and Hsp60 induces proteasome-mediated degradation of survivin, we examined survivin levels in siRNA-transfected SH-SY5Y cells treated with MG132 prior to cell lysis (Figure 3A). Ghosh et al. showed that Hsp60 binds to and stabilizes mitochondrial survivin [14]. As reported previously, we found that MG132 restored survivin expression in Hsp60-deficient cells (Figure 3A, lane 4 vs. lane 9), indicating that Hsp60 depletion induces degradation of survivin via the proteasome. By contrast, expression of survivin in CCAR2-deficient cells recovered slightly, but not completely, following treatment with MG132 (Figure 3A, lane 3 vs. lane 8), indicating that CCAR2 is not involved directly in survivin stabilization. To rule out the possibility that destabilization of survivin in CCAR2- and Hsp60-deficient cells occurs via lysosomes, we treated SH-SY5Y cells with chloroquine, an inhibitor of lysosome-mediated degradation, prior to cell lysis (Figure 3B). Chloroquine induced accumulation of LC3-II, a marker of lysosomal inhibition. Downregulation of survivin was not reproduced by chloroquine, indicating that siRNAs targeting CCAR2 and Hsp60 do not reduce survivin expression via lysosome-mediated degradation (Figure 3B). Next, we examined the levels of survivin mRNA in CCAR2- and Hsp60-deficient SH-SY5Y cells. Depleting CCAR2 or Hsp60 resulted in low survivin expression at the mRNA level (Figure 3C). Downregulation of survivin mRNA via depletion of CCAR2 and Hsp60 was confirmed using two different siRNAs (Figure 3D). Overall, the above experiments demonstrate that deficiency in CCAR2 and Hsp60 downregulates expression of survivin mRNA, ultimately downregulating expression of survivin protein. Downregulation of survivin mRNA expression is probably due to upregulation of p53 (Figure 3A), a negative regulator of survivin transcription (see Discussion section) [38]. The level of p53, which undergoes proteolytic degradation in many cells, was unchanged in SH-SY5Y cells treated with MG132, a finding consistent with that reported in previous studies (Figure 3A, lane 1 vs. lane 6) [39]. However, p53 was upregulated in CCAR2- and Hsp60-deficient cells (Figure 3A, lanes 3, 4, and 5), which may sensitize these cells to apoptotic stimuli [11]. Upregulation of p53 in Hsp60-deficient cells is consistent with the findings of a previous study showing that transfection of Hsp60 siRNA increases the level of mitochondrial p53 [14]. To confirm whether p53 is one of the factors that regulate survivin expression in CCAR2- and Hsp60-deficient cells, we compared expression of survivin protein in SH-SY5Y (p53 wild-type and MYCN non-amplified cells) and BE(2)-M17 (p53 mutant type and MYCN amplified cells) neuroblastoma cells [40,41,42]. p53 was upregulated in CCAR2- and Hsp60-deficient SH-SY5Y cells, but not in BE(2)-M17 cells. While the level of survivin protein was downregulated in both CCAR2- and Hsp60-deficient SH-SY5Y cells, it was downregulated only in Hsp60-deficient BE(2)-M17 cells. Taken together with Figure 2 and Figure 3, these data indicate that CCAR2-mediated survivin expression is primarily dependent on negative transcription mediated by p53, and that Hsp60-mediated survivin expression is dependent on its role in survivin stabilization as well as p53-dependent negative transcription. Although the mechanism by which CCAR2 and Hsp60 maintain survivin expression requires further investigation, the data suggest that both proteins are key players in survivin regulation.

### 2.4. Expression of CCAR2 and Hsp60 in Patients with Neuroblastoma Correlate Positively with that of Survivin

**Figure 4:**
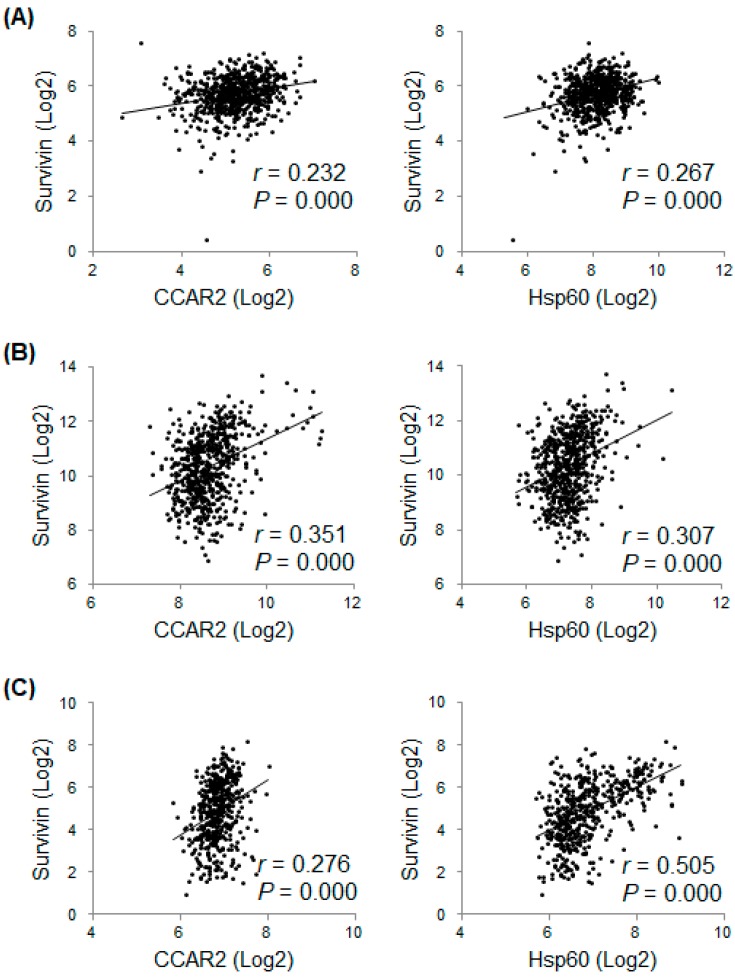
Expression of survivin mRNA in human cancer cell lines and neuroblastoma tissues shows a positive correlation with that of CCAR2 and Hsp60. Expression of mRNAs encoding CCAR2, Hsp60, and survivin was downloaded from a publicly available database that included the data from commonly used human cancer cell lines (E-MTAB-2706, *n* = 675) (**A**), neuroblastoma tumors (GSE45547, *n* = 649) (**B**), and neuroblastoma tumors (GSE62564, *n* = 498) (**C**). Expression of each mRNA value was Log2-transformed. Pearson’s correlation analysis was used to examine the relationship between each mRNA. *r*, Pearson’s correlation coefficient; *P*, *P*-value.

The above data demonstrate that CCAR2 and Hsp60 are required for expression of survivin; therefore, it is of interest to examine the correlation between expression of CCAR2, Hsp60, and survivin in vivo. First, we examined expression of mRNAs in 675 commonly used human cancer cell lines (data obtained from the ArrayExpress dataset E-MTAB-2706). Expression of CCAR2 and Hsp60 showed a weak positive relationship (0.2 < r < 0.4) with that of survivin (Figure 4A). Next, we examined the correlation between CCAR2, Hsp60, and survivin in 649 and 498 neuroblastoma tissues (data obtained from GEO datasets GSE45547 and GSE62564, respectively). The results revealed that expressions of both CCAR2 and Hsp60 showed a weak (0.2 < r < 0.4) or moderate (0.4 < r < 0.6) positive relationship with that of survivin (Figure 4B,C). This implies that CCAR2 and Hsp60 play a role in survival of tumor cells, possibly by upregulating survivin.

### 2.5. Expression of CCAR2, Hsp60, and Survivin Shows a Negative Correlation with Survival of Neuroblastoma Patients

**Figure 5:**
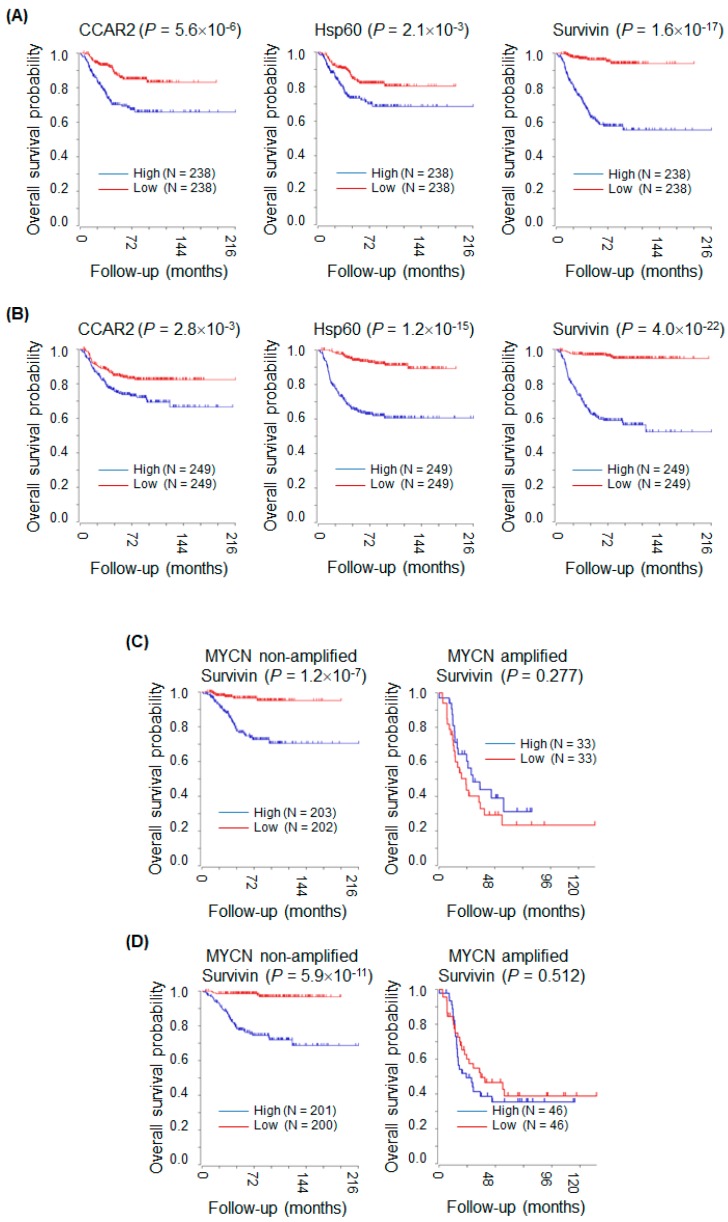

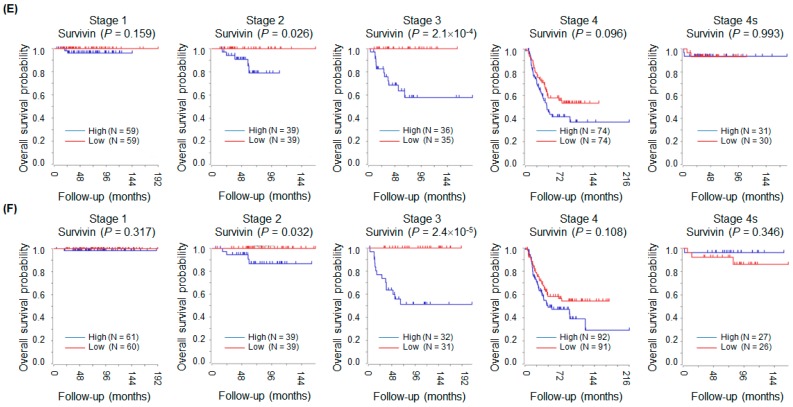
High expression of CCAR2, Hsp60, and survivin is associated with poor survival of neuroblastoma patients. Kaplan–Meier survival analysis was performed using the R2 platform. Expression of CCAR2, Hsp60, and survivin was defined as high (above the median value) or low (below the median value). *p*-values were calculated using the log-rank test. (**A**) Neuroblastoma patients (*n* = 476) from the Kocak dataset. (**B**) Neuroblastoma patients (*n* = 498) from the SEQC-RPM dataset. (**C**,**D**) The Kocak (**C**) and SEQC-RPM (**D**) datasets were sub-grouped into MYCN non-amplified and MYCN amplified subsets. (**E**,**F**) The Kocak (**E**) and SEQC-RPM (**F**) datasets were sub-grouped according to neuroblastoma stage.

The above data suggest that both CCAR2 and Hsp60 promote tumor cell survival by upregulating expression of survivin. This implies that tumors expressing high levels of CCAR2 and Hsp60 would be more aggressive because survivin is anti-apoptotic. Here, we used the R2 platform to examine the association between expression of mRNA encoding CCAR2, Hsp60, and survivin and survival of neuroblastoma patients. In the Kocak dataset [43], only 476 (for whom survival data were available) out of 649 neuroblastoma patients were used for Kaplan–Meier analysis. High levels of CCAR2, Hsp60, and survivin mRNA were associated with poor overall survival (Figure 5A). In addition, results from the SEQC-RPM dataset [44] (498 neuroblastoma patients) showed that high expression of these molecules was associated with poor overall survival (Figure 5B). Next, the association between survivin expression and overall survival of neuroblastoma patients was analyzed according to MYCN amplification and stage. Data from both the Kocak and SEQC-RPM datasets revealed that high expression of survivin was associated with poor overall survival of neuroblastoma patients with non-amplified MYCN, but not of those with amplified MYCN (Figure 5C,D). Next, the Kocak and SEQC-RPM datasets revealed that high expression of survivin was associated with poor overall survival of neuroblastoma patients in Stage 2 and 3, but not in Stage 1, 4, and 4s (Figure 5E,F) [45]. Overall, survivin would be a useful biomarker and prognostic marker in neuroblastoma depending on MYCN amplification and stage.

## 3. Discussion

Here, we show that both CCAR2 and Hsp60 are required for maintenance of survivin expression. Furthermore, expression of mRNA encoding CCAR2 and Hsp60 correlates positively with that of survivin in neuroblastoma tissues. High expression of each mRNA was associated with poor survival of neuroblastoma patients, suggesting that tumor cells with high expression proliferate more rapidly. Taken together, the results demonstrate that CCAR2 and Hsp60 act as pro-survival factors in neuroblastoma.

Survivin exerts its anti-apoptotic function in the cytosol and mitochondria. First, following apoptotic stress, survivin is released from the mitochondria into the cytosol, where it interacts with HBXIP (Hepatitis B X-interacting protein) [46] and XIAP (X-linked inhibitor of apoptosis protein) [47], thereby inhibiting caspase and conferring cytoprotection. Survivin interacts with SMAC/DIABLO and antagonizes its pro-apoptotic activity in the cytosol [48]. However, cells that express survivin only in the cytosol are not protected from apoptotic stimuli, indicating that targeting of survivin to the mitochondria is a prerequisite for apoptosis inhibition [49]. Second, the anti-apoptotic function also occurs in mitochondria. Survivin sequesters SMAC/DIABLO in mitochondria and prevents its release, thereby inhibiting apoptosis [50]. In summary, the mitochondrial localization of survivin is very important for its anti-apoptotic function.

Ghosh et al. showed that Hsp60 stabilizes mitochondrial survivin [14]. Cohen-Sfady et al. also demonstrated that treatment of B cells with recombinant Hsp60 upregulates survivin and protects cells from apoptosis [51]. The significance of CCAR2-Hsp60 complex in survivin expression is still undefined. Based on the previous reports that CCAR2 interacts with Hsp60 [11], and that Hsp60 stabilizes survivin in mitochondria [14], our data suggest that CCAR2 sequesters survivin in the mitochondria and brings survivin to Hsp60 to ensure stabilization (Figure 1A,B). The suggestion that CCAR2 is a core protein for Hsp60-dependent survivin stabilization is supported by Figure 1C. The interaction between CCAR2, Hsp60, and survivin was similar in control and Hsp60-deficient cells; in other words, the relative extent of their interaction increased in Hsp60-deificient cells (Figure 1C). Considering our previous reports that interaction between CCAR2 and Hsp60 increased following treatment of rotenone [11], this might be due to an increase in the binding affinity of CCAR2 for the remaining survivin and Hsp60 in response to mitochondrial stress induced by Hsp60 depletion. The enhanced binding would contribute to survivin stabilization to cope with mitochondrial stress. However, while MG132 restored expression of survivin in Hsp60-deficient cells (Figure 3A, lane 4 vs. lane 9), it did not restore survivin levels in cells lacking both CCAR2 and Hsp60 (Figure 3A, lane 5 vs. lane 10). It suggests that another regulatory mechanism, yet to be identified, controls survivin expression.

The data presented herein demonstrate that Hsp60 is not only a key regulator of survivin protein stability, but also a regulator of survivin mRNA expression, although the latter probably occurs via an indirect pathway. Expression of survivin mRNA is regulated by several factors [52,53]. The survivin promoter does not have a TATA box and possesses GC-rich sequences. Expression of the survivin gene increases during G2/M, a process controlled by the cell cycle-dependent element/cell cycle gene homology region (CDE/CHR). In addition, the survivin promoter also contains numerous binding sites for transcription factors such as Sp1, GATA-1, NF-κB, STAT3, DEC1, KLF5, HIF-1α, and E2F1, which induce survivin expression. By contrast, survivin expression is repressed by p53 [38]. However, considering that CCAR2 and Hsp60 are located in the mitochondria, it is less likely that they regulate expression of survivin mRNA directly. Instead, as shown in Figure 3A,C, cells harboring wild-type p53 showed increased levels of p53 protein and decreased levels of survivin mRNA when they were depleted of CCAR2 and Hsp60. By contrast, cells harboring a p53 mutation did not downregulate survivin protein under conditions of CCAR2 deficiency (Figure 3E). Hsp60 deficiency downregulated survivin protein in cells harboring a p53 mutation because Hsp60 is still required for stabilization of the survivin protein. Although we have not yet checked the levels of survivin mRNA in CCAR2- and Hsp60-deficient cells harboring a p53 mutation, it suggests that both CCAR2 and Hsp60 are regulatory factors for expression of survivin mRNA in a p53-dependent manner. Furthermore, cytosolic Hsp60 transactivates NF-κB-dependent genes such as MnSOD, as well as pro-inflammatory cytokines and chemokines, in response to TNF-α [54,55], suggesting a role for Hsp60 as a transcriptional regulator. CCAR2 also controls activity of several transcription factors (see Introduction section) [1,3]. Therefore, it would be interesting to investigate whether other transcription factors responsible for survivin expression are regulated by CCAR2 and Hsp60.

Overall, survivin is regulated by CCAR2 and Hsp60 in two different modes. Once survivin is translated and translocated to mitochondria, it is stabilized by Hsp60, which blocks the release of survivin from mitochondria to cytosol and its proteasomal degradation. Considering that CCAR2 binds survivin in the absence of mitochondrial stress (Figure 1) and that that the formation of CCAR2-Hsp60 complex increases following mitochondria stress [11], a possible model is that CCAR2 sequesters survivin and brings it to Hsp60 in mitochondria. It means that CCAR2-Hsp60 complex is required for expression of survivin at the protein level. In addition, survivin is regulated by CCAR2 and Hsp60 at the mRNA level as well, although it is regulated in an indirect manner as discussed in the previous paragraph. We already reported that both CCAR2 and Hsp60 are required for maintenance of mitochondrial membrane potential [11]. The loss of mitochondrial homeostasis in CCAR2- and Hsp60-deficient cells might provoke apoptotic signaling such as p53 upregulation [56]. p53 acts as a negative transcriptional factor for expression of survivin in nucleus under the conditions that CCAR2 and Hsp60 are deficient. Furthermore, these data also support our previous findings that downregulation of survivin, an anti-apoptotic factor, and upregulation of p53, a pro-apoptotic factor, renders CCAR2- and Hsp60-deficient SH-SY5Y cells more sensitive to mitochondrial stress [11].

CCAR2 is overexpressed by several cancers, and expression is related to prognosis [2]. Most cancers express high levels of Hsp60, which may correlate with tumor cell growth [24]. Upregulation of survivin expression is associated with poor survival; therefore, survivin is considered a potential therapeutic target [57]. Our data demonstrate that high expression of CCAR2, Hsp60, and survivin is predictive of a poor prognosis for human neuroblastoma patients (Figure 5A,B). In particular, survivin is a prognostic marker in neuroblastoma patients with non-amplified MYCN (Figure 5C). Amplification of MYCN is the best characterized marker of high risk in neuroblastoma [58]. The importance of CCAR2-Hsp60-survivin network is supported by our findings that expression of survivin was dependent on both CCAR2 and Hsp60 in MYCN non-amplified SH-SY5Y cells (Figure 3E). In addition, our results demonstrate that survivin is associated with a poor prognosis in neuroblastoma patients in Stage 2 and 3, but not in Stage 1, 4, and 4s. This implies that survivin can be a prognostic indicator in neuroblastoma patients whose tumors are not removed surgically but have not metastasized to distant lymph nodes and organs. However, the relationship between survivin expression and MYCN amplification and stage of neuroblastomas requires further analysis.

In summary, CCAR2 and Hsp60 act as pro-survival factors by upregulating survivin expression. The results also suggest that CCAR2 and Hsp60, as well as survivin, are useful therapeutic targets for cancer.

## 4. Materials and Methods

### 4.1. Cell Culture

SH-SY5Y and BE(2)-M17 human neuroblastoma cells established from metastatic bone marrow were maintained in DMEM and DMEM/F-12, respectively, supplemented with 10% FBS, 100 U/mL penicillin G sodium, 100 μg/mL streptomycin sulfate, and 0.25 μg/mL amphotericin B. Cells were incubated at 37 °C in 5% CO_2_ incubator.

### 4.2. Small Interfering RNA (siRNA) Transfection

Universal, CCAR2, and Hsp60 siRNAs were synthesized by ST Pharm. Co., LTD. (Seoul, Korea). The siRNA duplexes were as follows: universal (control) siRNA, AUGAACGUGAAUUGCUCAAdTdT; CCAR2 (NM_021174) siRNA #1, CAGCUUGCAUGACUACUUUdTdT; CCAR2 siRNA #2, CAGCGGGUCUUCACUGGUAdTdT; Hsp60 (NM_002156) siRNA #1, UGAAGAAAUUGCACAGGUUdTdT; Hsp60 siRNA #2, UGAAUGAACGGCUUGCAAAdTdT. Transfection was performed with 20 nM siRNA using Lipofectamine RNAiMax (Invitrogen, Carlsbad, CA, USA). Forty-eight hours after transfection, all experiments were performed.

### 4.3. Immunoprecipitation and Western blotting

Cells were lysed using NETN lysis buffer (100 mM NaCl, 1 mM EDTA, 20 mM Tris-HCl, 0.5% Nonidet P-40, 50 mM β-glycerophosphate, 10 mM NaF, and 1 mM Na_3_VO_4_) containing a protease inhibitor cocktail (535140, Millipore, Burlington, MA, USA) on ice for 10 min. After centrifugation at 12,000× *g* for 5 min, the supernatant was saved as whole cell lysates. For the immunoprecipitation, the whole cell lysates were incubated with rabbit IgG (ab27478, Abcam, Cambridge, UK), anti-survivin (NB500-201, Novus Biologicals, Centennial, CO, USA) or anti-CCAR2 antibody (hoemade [7] or H00057805-D01, Abnova, Taipei City, Taiwan), and Protein A sepharose 4 Fast Flow (17-5280-01, GE Healthcare, Chicago, IL, USA). The pull-down complexes were boiled with Laemmli buffer at 95 °C for 5 min and then loaded onto SDS-polyacrylamide gel. Western blotting was performed following a routine protocol [59]. The antibodies used for western blotting were as follows: β-actin (4970, Cell Signaling, Danvers, MA, USA), CCAR2 (homemade [7] or H00057805-D01P, Abnova, Taipei City, Taiwan), cytochrome c (sc-13156, Santa Cruz Biotechnology, Dallas, TX, USA), GAPDH (sc-25778, Santa Cruz Biotechnology, Dallas, TX, USA), Hsp60 (sc-59567, Santa Cruz Biotechnology, Dallas, TX, USA), Hsp90 (2D11B9, Enzo Life Sciences), LC3 (PM036, MBL International Corp., Woburn, MA, USA), p53 (sc-126, Santa Cruz Biotechnology, Dallas, TX, USA), and survivin (NB500-201, Novus Biologicals, Centennial, CO, USA).

### 4.4. Subcellular Fractionation

The crude mitochondrial fraction was prepared as described [11,60]. Briefly, the cell pellet was resuspended in ice-cold buffer (225 mM mannitol, 75 mM sucrose, 30 mM Tris-HCl pH 7.4, and 0.1 mM EGTA) and vortexed for 30 s. After centrifugation at 600× *g* at 4 °C for 5 min, the supernatant was centrifuged again under the same conditions. The supernatant was then centrifuged at 7000× *g* at 4 °C for 10 min. The final supernatant was the cytosolic fraction, containing lysosomes and microsomes. The pellet containing mitochondria was washed to remove cytosolic residues. It was then resuspended in ice-cold buffer (225 mM mannitol, 75 mM sucrose, and 30 mM Tris-HCl pH 7.4) and centrifuged again at 10,000× *g* for 10 min at 4 °C. To avoid disruption of the mitochondria, the mitochondrial suspension was transferred using a wide-bore tip. The crude mitochondrial pellet from the last centrifugation was resuspended in ice-cold buffer (250 mM mannitol, 5 mM HEPES pH 7.4, and 0.5 mM EGTA). GAPDH and Hsp90 are cytosolic markers, and cytochrome c is a mitochondrial marker.

### 4.5. Reverse Transcription-Polymerase Chain Reaction (RT-PCR)

Total RNA was isolated using Trizol reagent (Invitrogen, Carlsbad, CA, USA) and was used to synthesize cDNA using PrimeScript^TM^ reverse transcriptase (Takara Bio Inc., Shiga, Japan). The sequences of each forward (F) and reverse (R) primer used for PCR were as follows: β-actin-F, GCTCGTCGTCGACAACGGCT; β-actin-R, CAAACATGATCTGGGTCATCTTCTC; CCAR2-F, CAAACATCCCACACACTTCAC; CCAR2-R, GACCTGGATCCGGCTTGGATG; Hsp60-F, CCCACAGTCTTTCGCCAGAT; Hsp60-R, CTTGGCTATAGAGCGTGCCA; survivin-F, GCATGGGTGCCCCGACGTTG; survivin-R, GCTCCGGCCAGAGGCCTCAA.

### 4.6. Analysis of Correlation between Gene Expressions

Microarray data are available in the ArrayExpress database (www.ebi.ac.uk/arrayexpress) (access on 14 June 2017) (Accession No. E-MTAB-2706) and the National Center for Biotechnology Information (NCBI) Gene Expression Omnibus (GEO) database (Accession No. GSE45547 [43] and GSE62564 [44]). Publically available raw files were downloaded, and transcript values for CCAR2 (KIAA1967), Hsp60 (HSPD1), and survivin (BIRC5) were Log2-transformed. Pearson’s correlation coefficient (*r*) was calculated to determine the correlation between the two transcripts.

### 4.7. Analysis of the Association between Gene Expression and Survival of Neuroblastoma Patients

Expressions of CCAR2 (KIAA1967), Hsp60 (HSPD1), and survivin (BIRC5) mRNA were examined in neuroblastoma tissues of 476 (Kocak dataset) [43] and 498 (SEQC-RPM dataset) [44] human neuroblastoma patients from the publicly available gene expression datasets (downloaded from R2: Genomics Analysis and Visualization Platform [http://r2.amc.nl]) (access on 13 June 2017). The median value for each transcript was used as the cut-off point, and expression was defined as high (above the median value) or low (below the median value). The association between transcript levels and overall survival was visualized using Kaplan–Meier curves, and the significance of differences was assessed using the log-rank test [61].

## Abbreviations

CCAR2	Cell cycle and apoptosis regulator 2
Hsp60	Heat shock protein 60
